# Traumatic Orbital Compartment Syndrome in Children—Systematic Review and Descriptive Summary

**DOI:** 10.3390/children13080982

**Published:** 2026-07-24

**Authors:** Tobias Jhala, Lea Berger, Jonathan Aichner, Markus Lehner, Justus Lieber, Jörg Fuchs

**Affiliations:** 1 Department of Pediatric Surgery and Pediatric Urology, University Children’s Hospital Tübingen, 72076 Tuebingen, Germany; 2 Department of General, Visceral and Transplantation Surgery, Tübingen University Hospital, 72076 Tuebingen, Germany; lea.berger@kliniken-rt.de; 3 Department of Paediatric Urology, Great Ormond Street Hospital for Children, London WC1N 3JH, UK; jonathan.aichner@gosh.nhs.uk; 4 Department of Pediatric Surgery, Luzerner Kantonsspital, Spitalstrasse, 376004 Luzern, Switzerland; markus.lehner@luks.ch

**Keywords:** orbital compartment syndrome, orbital hematoma, subperiosteal hematoma, pediatric trauma, children, ocular trauma, retrobulbar hemorrhage, orbital trauma, vision loss

## Abstract

**Highlights:**

**What are the main findings?**

- Traumatic orbital compartment syndrome in children is predominantly associated with orbital subperiosteal hematoma and frequently occurs after minor blunt trauma.
- Pediatric orbital compartment syndrome appears to exhibit distinct clinical and pathophysiological characteristics compared with adult disease.

**What are the implications of the main findings?**

- Early recognition and timely decompression may be beneficial in preserving vision in children with orbital compartment syndrome.
- A pragmatic pediatric management approach considering age-specific injury patterns may support clinical decision-making in this rare emergency.

**Abstract:**

Topic: What are the epidemiology, mechanisms, management strategies, and visual outcomes of traumatic orbital compartment syndrome (OCS) in children? Clinical relevance: Traumatic OCS is a rare but vision-threatening emergency requiring rapid recognition and intervention. In children, diagnosis and management are challenging due to age-specific anatomy, limited cooperation, and heterogeneous trauma mechanisms. Current management strategies are largely extrapolated from adult data, and no pediatric-focused systematic synthesis is available. Methods: A systematic review was conducted in accordance with PRISMA guidelines. Embase, PubMed, and Web of Science were searched from inception to 10 December 2023, with an update on 9 October 2024 and 8 April 2025 using terms related to orbital compartment syndrome, orbital hematoma, trauma, and a validated pediatric search filter. Eligible studies included case reports and case series describing traumatic OCS in patients younger than 18 years. Three reviewers independently screened studies, extracted data, and assessed risk of bias using the Joanna Briggs Institute critical appraisal checklists for case reports and case series. Due to substantial heterogeneity, results were synthesized descriptively. The review protocol was registered prospectively in PROSPERO (CRD420251028845). Results: Of 1317 screened records, 90 studies were included, comprising 126 pediatric patients. Mean age was 10 years, with a male predominance (3.8:1). Falls and direct head trauma were the most common injury mechanisms. Subperiosteal hematoma was the predominant underlying pathology. Eleven patients had documented coagulopathies, frequently associated with extensive extracranial hematomas. Clinical presentation commonly included proptosis and visual symptoms, with onset either immediately after trauma or delayed by 4–7 days. Management strategies included observation (18/126), medical therapy (24/126), and surgical intervention (103/126; 70 as primary treatment). At a mean follow-up of 109 days, visual impairment was reported in 21 patients. Earlier surgical intervention was consistently associated with more favorable visual outcomes, although certainty of evidence was very low. Conclusions: Evidence limited to case reports and small case series suggests that pediatric OCS differs from adult disease with regard to epidemiology and underlying pathophysiology. Despite very low certainty of evidence, consistent patterns support a time-critical, clinically driven management approach. A pragmatic diagnostic and treatment algorithm is proposed to support early recognition and timely intervention in children with suspected OCS.

## 1. Introduction

Orbital compartment syndrome (OCS) is a rare but vision-threatening emergency that occurs when an increase in intraorbital pressure compromises perfusion to the optic nerve and retina [1]. It most commonly results from trauma. Awareness of OCS is of the upmost importance, since immediate recognition and intervention are critical, as irreversible visual loss can occur within 90 to 120 min of ischemia [2,3,4,5]. Therefore, clinical diagnosis remains paramount and should not be delayed by imaging [5,6,7]. Signs such as proptosis, vision loss, afferent pupillary defect, and a firm globe warrant immediate intervention [2,3]. The gold standard treatment in adults is lateral canthotomy with inferior cantholysis—a rapid bedside procedure that can preserve vision if performed promptly [2,8,9].

While OCS is well-documented in adults, its presentation in children and newborns is scarce [1,8,9,10]. There are important anatomical and physiological differences between adults and children, particularly regarding skull pliability and the adherence of tissue layers. These differences may result in distinct pathophysiological mechanisms and etiologies leading to OCS in pediatric patients [11,12,13,14,15,16].

To date, no systematic review complying with PRISMA guidelines has been carried out concerning OCS in children [13,17,18]. Given the potential devastating consequences of delayed diagnosis and treatment, this review aims to provide a more thorough understanding of the epidemiology and treatment options of orbital compartment syndrome in children.

## 2. Methods

### 2.1. Systematic Review Protocol

This study followed the Preferred Reporting Items for Systematic Reviews and Meta-analyses (PRISMA) reporting guidelines for systematic reviews and meta-analyses (PRISMA flowchart [19,20]. The study was registered on PROSPERO (ID: CRD420251028845).

### 2.2. Search Strategies

A systematic search was conducted including the following databases: PubMed, EMBASE and Web of Science Search terms included orbital emphysema, canthotomy, orbital compartment syndrome, orbital compression syndrome, retrobulbar hematoma, retrobulbar hemorrhage, subperiosteal orbital hematoma and trauma. Medical subject headings for each keyword were used. Furthermore, truncations on the keywords were used to expand the search results (see Supplementary Materials). A validated pediatric (defined as age <18 years) search filter for PubMed was used and adjusted according to the other databases’ search field tags [21]. No language restrictions were applied, and the searches were limited to human studies. The database searches were from the date of database inception to 8 April 2025.

### 2.3. Eligibility Criteria

PEOS was used to formulate eligibility criteria:

P (Population): Children (defined as age < 18 years).

E (Exposure): Patients suffering from traumatic orbital compartment syndrome.

O (Outcomes): Injury pattern, clinical presentation, treatment and functional outcome.

S (Studies): Due to the anticipated limited primary data, all types of studies, except systematic reviews, were included. Studies that include both adults and children but present data in a pooled manner, not specifying the functional outcomes of the included children, were excluded.

### 2.4. Study Selection, Data Abstraction and Quality Appraisal

After the removal of duplicates, two reviewers screened the search results and applied the eligibility criteria listed above to identify relevant articles, with a third reviewer resolving conflicts concerning the inclusion of articles. Articles that were potentially relevant were sought for retrieval and included in the full-text review. The review process mentioned above was also applied for the full-text screening. The following data were extracted from the articles: date of publication, methodology, number of cases, age of children, diagnosis, injury pattern, symptoms, time to symptom onset, time to presentation, clinical findings, associated injuries and diagnosis, diagnostic work-up, treatment, and visual outcome.

The quality of articles was assessed by two independent reviewers. The Joanna Briggs checklist for case reports and case series was used to evaluate the eligible articles. Article quality was classified according to the percentage of criteria they met on the Joanna Briggs checklist (good = >80%; fair 50–79%; poor < 50%) [22]. If the quality was deemed poor or fair, the study was excluded; however, no case report or case series was deemed poor or fair, and therefore no exclusion had to be made on the basis of study quality.

Furthermore, visual outcomes were harmonized using the information provided in the original reports and categorized according to WHO definitions whenever possible. However, visual acuity testing methods were inconsistently reported, particularly in infants and young children, limiting comparability across age groups.

### 2.5. Statistical Analysis

A qualitative analysis was done concerning epidemiological parameters of traumatic orbital compartment syndrome in children. Relevant metrics, like age, eye involvement, trauma mechanism, direct pathomechanism leading to OCS, time to symptom onset, time to presentation, diagnostics and treatments, as well as outcomes, were pooled and presented in a narrative review. Subgroups were detected and also presented using a narrative review. Owing to the sparse and heterogeneous nature of the dataset, subgroup analyses were conducted but the results have to be considered exploratory. A quantitative analysis was undertaken for subgroup comparison concerning time to symptoms, time to consultation, eye involvement and associated injuries and diagnosis. Welch test was used to test for statistical significance between subgroups in case of ordinal variables (e.g., comparison of symptom onset). The Chi square test was performed to test for differences between subgroups in case of nominal variables (e.g., difference in prevalence of coagulopathy) [23,24]. Statistical analysis was performed using Microsoft Excel 365 (Microsoft Corporation, Redmond, WA, USA).

## 3. Results

### 3.1. Study Selection and Characteristics

**Figure 1:**
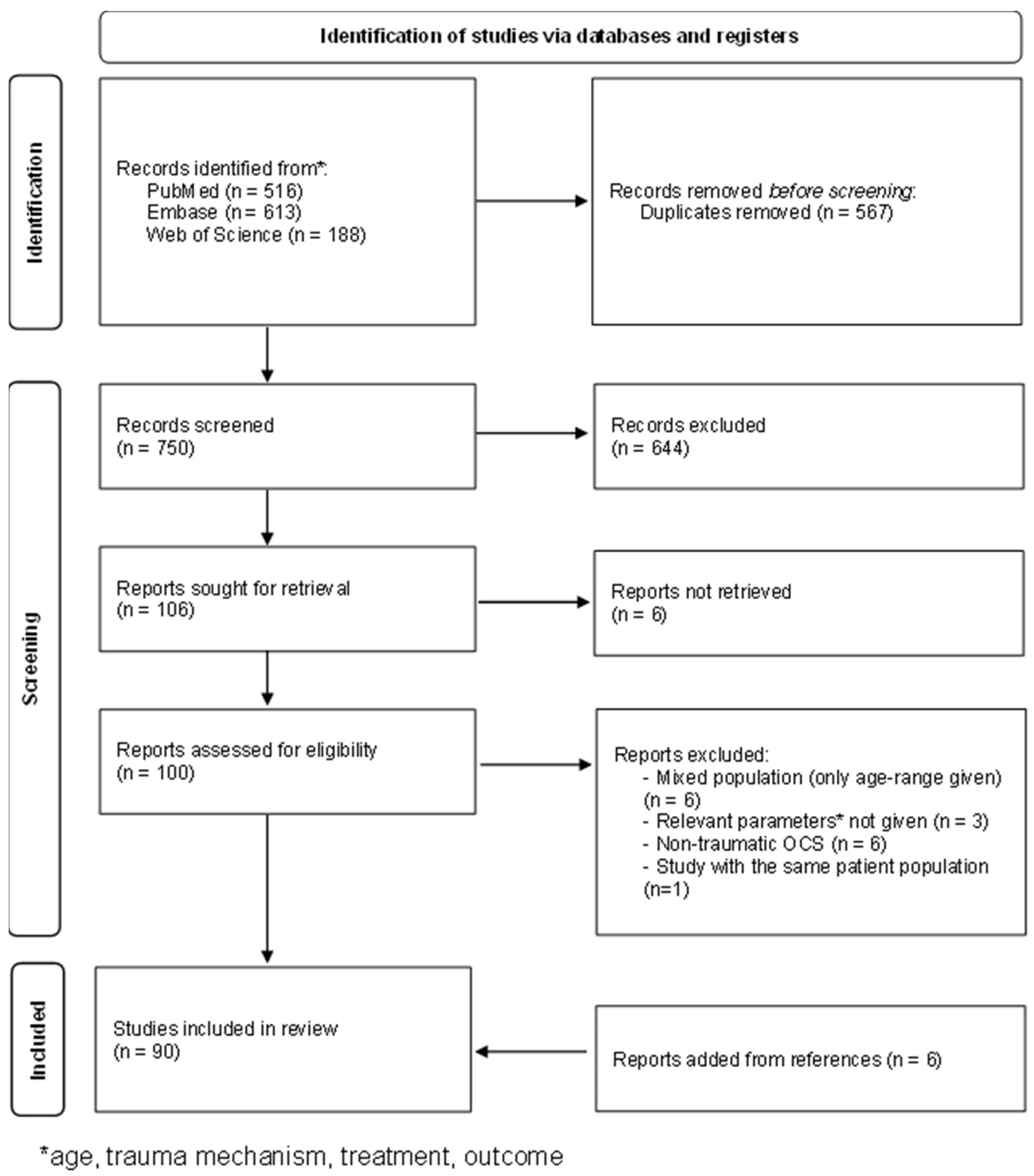
PRISMA Flowchart.

A total of 1317 results were generated from the initial search. Following the outlined screening process, 90 studies were identified as eligible for inclusion in the analysis [1,3,7,9,10,11,12,13,14,15,17,18,25,26,27,28,29,30,31,32,33,34,35,36,37,38,39,40,41,42,43,44,45,46,47,48,49,50,51,52,53,54,55,56,57,58,59,60,61,62,63,64,65,66,67,68,69,70,71,72,73,74,75,76,77,78,79,80,81,82,83,84,85,86,87,88,89,90,91,92,93,94,95,96,97,98,99,100,101,102]. These studies consisted primarily of case reports and case series. A total of 126 patients (138 affected eyes) were extracted for analysis (Figure 1). A detailed list of the included studies is provided in the Supplementary Materials.

### 3.2. Epidemiology of OCS

The mean age of the 126 children was 10 years (standard deviation = 4.7). The male-to-female ratio was 3.8:1 (100 males:26 females). The right eye was affected in 55 patients, the left eye in 59 patients, and 12 patients exhibited bilateral orbital compartment syndrome. During analysis, three distinct subgroups and one special patient population were identified: patients with associated epidural hematoma (EDH; 21 patients), patients with associated subgaleal hematoma (SGH; 25 patients) and patients with no associated extraorbital hematoma (NOA; 72 + 8 patients). Newborns formed a distinct group, which is discussed separately below. For the statistical comparison of the subgroups, the eight newborns were included in the cohort of patients without associated extraorbital hematoma.

### 3.3. Trauma Mechanism and Underlying Pathology Leading to OCS

**Figure 2:**
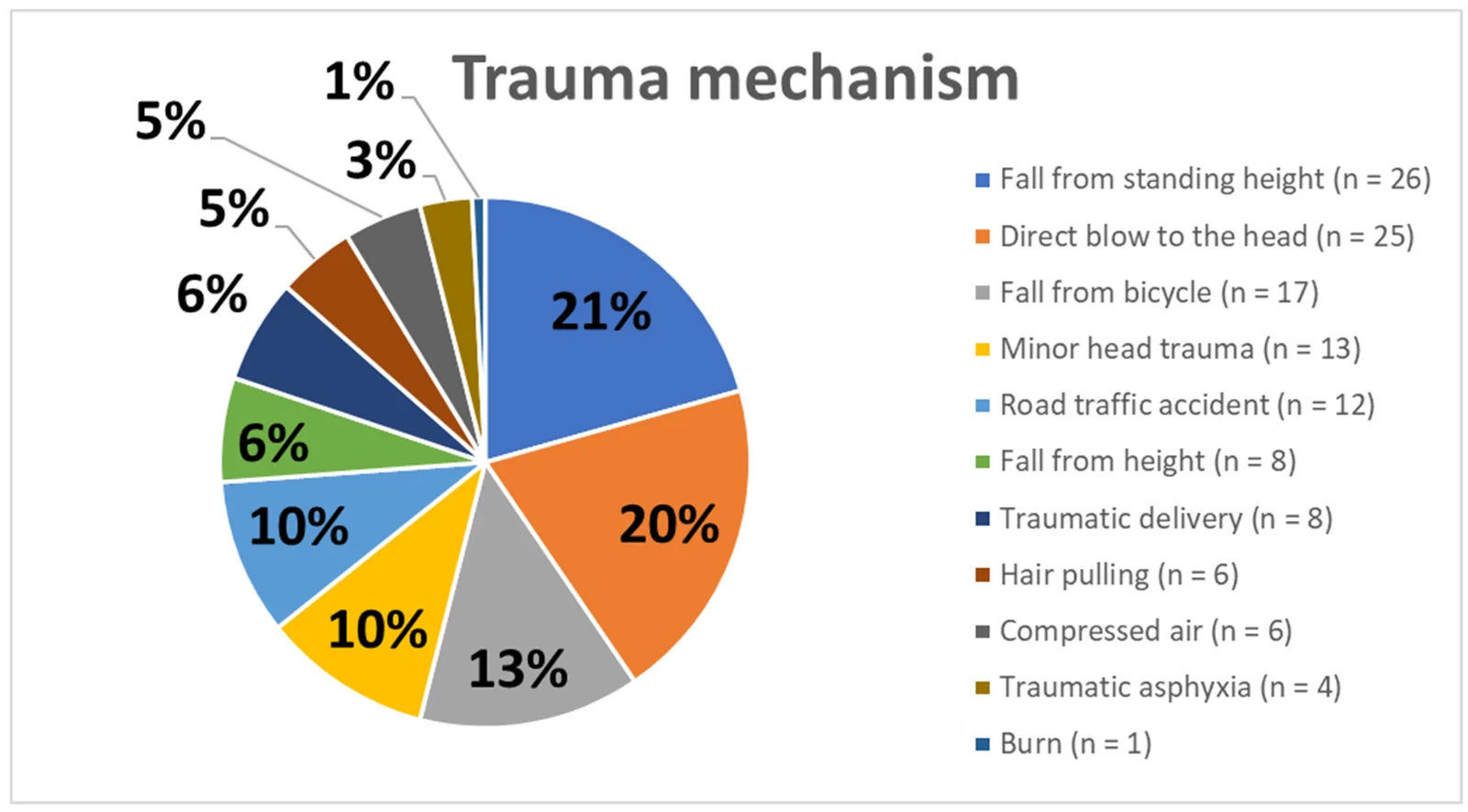
Reported trauma mechanism.

**Figure 3:**
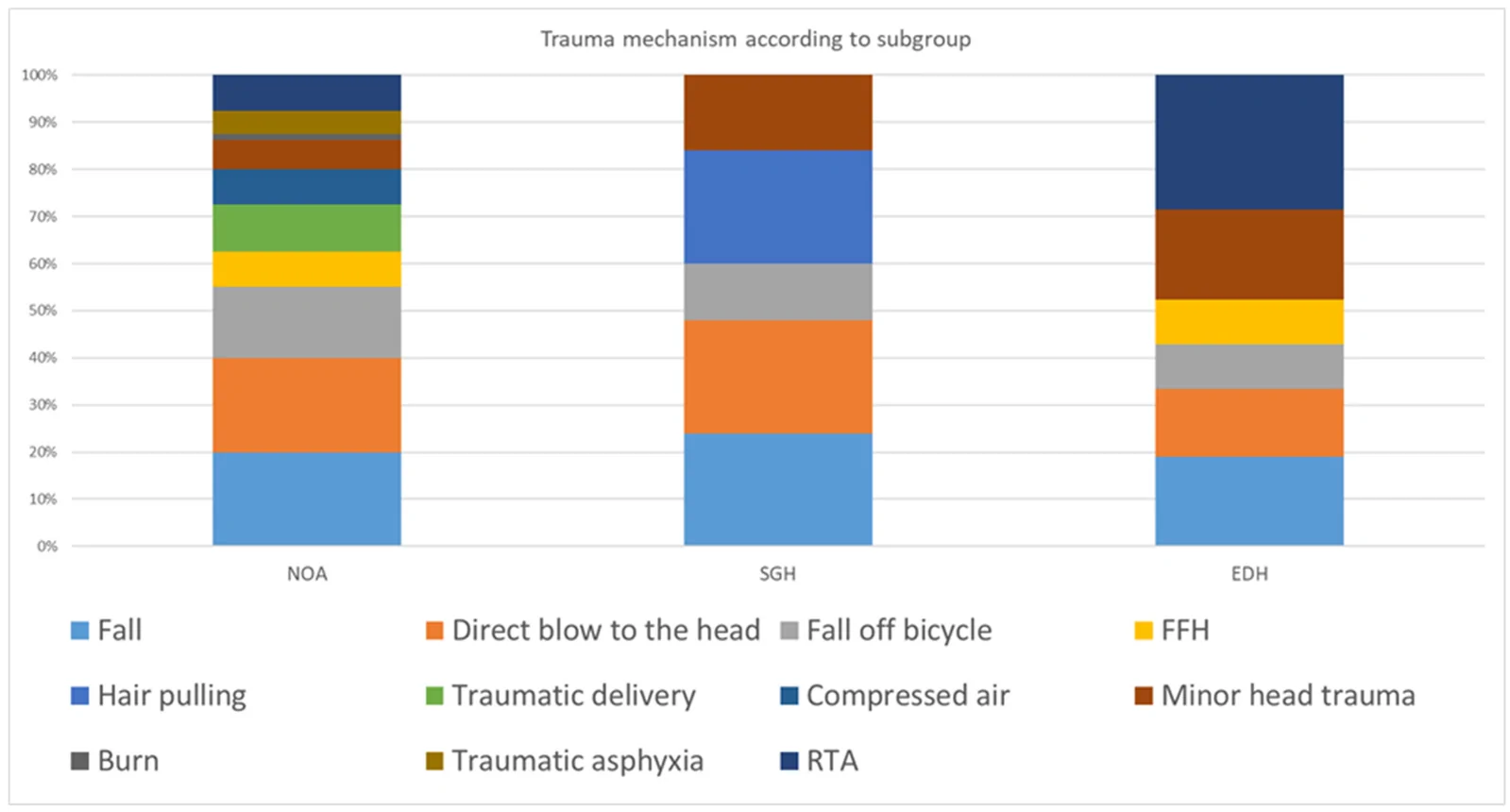
Trauma mechanism concerning subgroups. RTA = road traffic accident; FFH = fall from height.

The reported traumatic events leading to OCS were categorized into eleven distinct groups (Figure 2). The most common mechanisms were falls from standing height (21%) and direct blows to the head (20%). Several trauma mechanisms had a strong association with the above-mentioned subgroups (Table 1). All newborns (included in the NOA group in Table 1) developed OCS because of traumatic delivery. Hair-pulling was only associated with OCS in the context of SGH. Burn and compressed air had no association with SGH or EDH, while RTAs were causative for approximately 30% of OCS in association with EDH (Figure 3).

**Figure 4:**
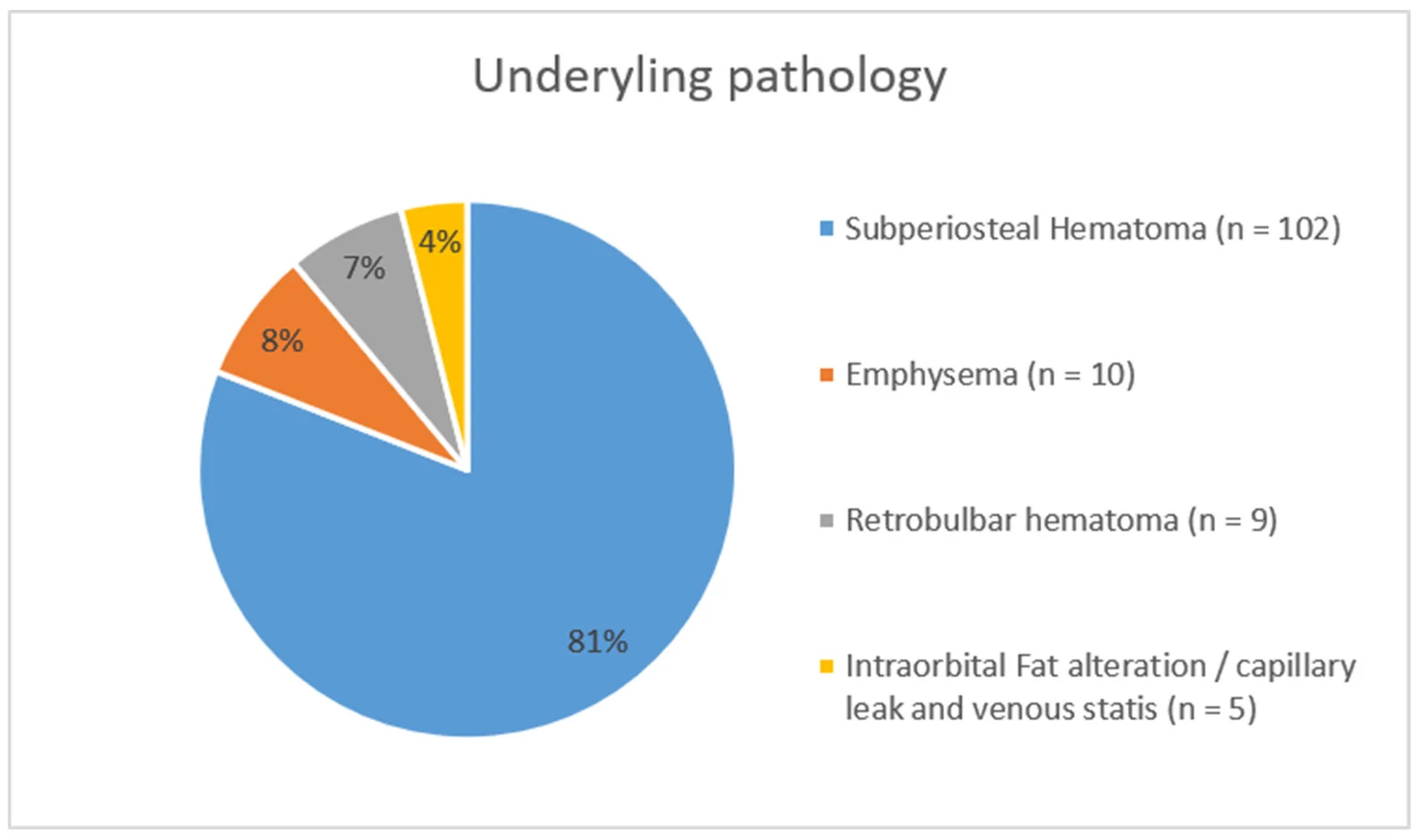
Underlying pathomechanism responsible for OCS development, detailed according to subgroups. OCS = orbital compartment syndrome.

Diagnostic evaluations revealed several underlying pathophysiological mechanisms responsible for the development of OCS, with subperiosteal hematoma being the most common (81%) (Figure 4 and Table 1).

Orbital emphysema occurred following compressed air injuries (e.g., air compressor pistol injury), direct head trauma, or falls from bicycles. Intraorbital fat alterations and venous stasis were associated with traumatic asphyxia or burns, as noted above.

### 3.4. Associated Injuries, Intracranial Bleeding, and Coagulopathies

A total of 30 patients had additional fractures involving either the viscerocranium or neurocranium. Of these, 27 fractures involved the viscerocranium, specifically the orbital walls. The orbital roof was affected in 15 patients, followed by the orbital floor in 4 patients, and the medial and lateral orbital walls in 2 patients each. Orbital fractures unspecified in type were present in 4 patients. No statistically significant correlation was observed between orbital fractures and subperiosteal hematoma (*p* = 0.31). However, fractures were significantly more prevalent in patients with associated epidural hematoma than in those with subgaleal hematoma or no hematoma (*p* < 0.05).

Eleven patients had an underlying coagulopathy, four of whom were previously diagnosed prior to the onset of OCS. Nine of these patients had congenital coagulopathies, including sickle cell disease, Hemophilia A or B, hypofibrinogenemia, and deficiencies of factors VII, VIII, IX, and XIII. Two patients had iatrogenic coagulopathies: one was receiving aspirin following a liver transplantation, and the other was undergoing ECMO (extracorporeal membrane oxygenation) therapy at the time of OCS development. A significant association was found between coagulopathy and the development of subgaleal hematoma (*p* < 0.05). Moreover, a significant correlation was observed between bilateral eye involvement and the presence of subgaleal hematoma (*p* < 0.05).

### 3.5. Symptoms and Time to Consultation

**Figure 5:**
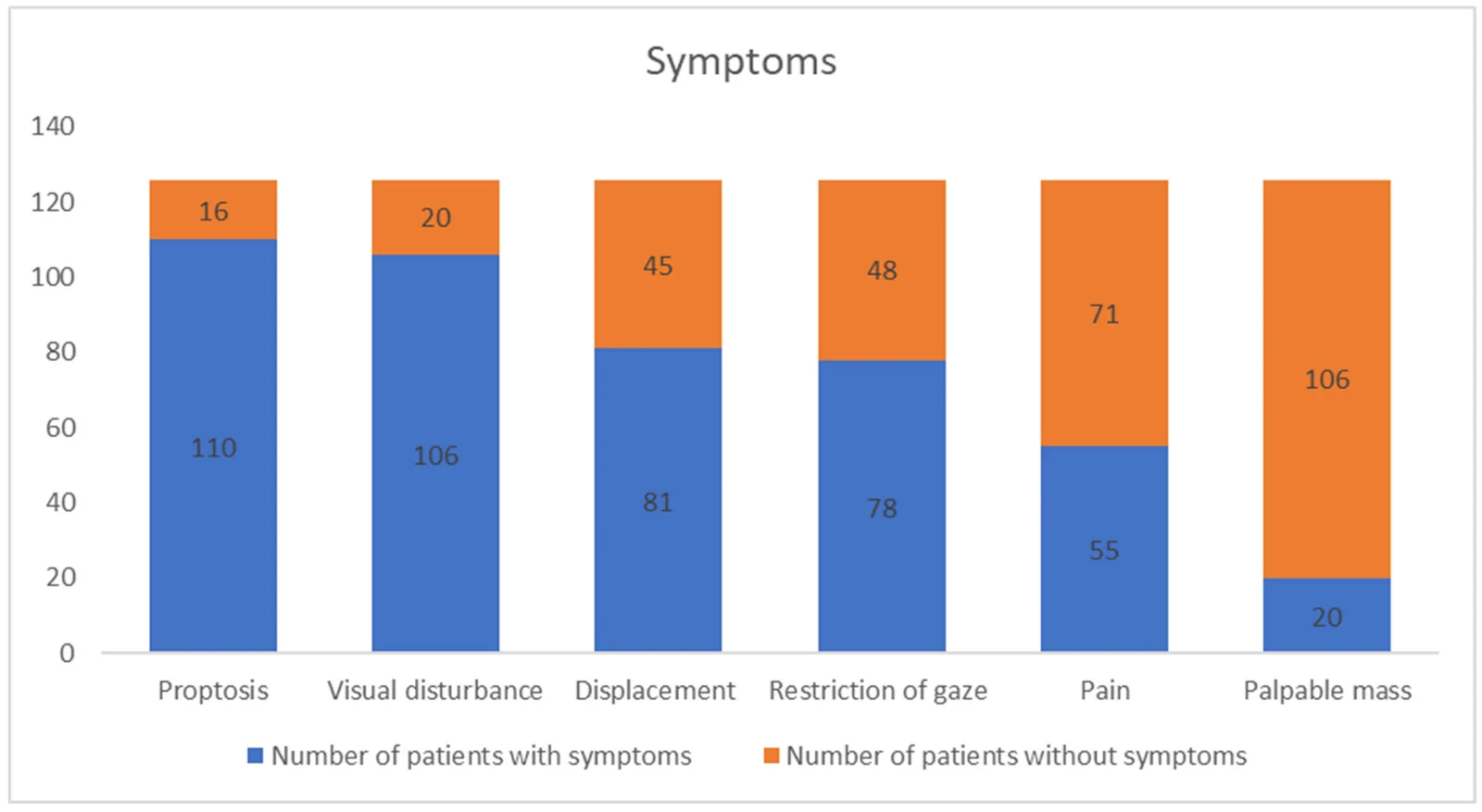
Prevalence of symptoms of patients affected by OCS.

**Figure 6:**
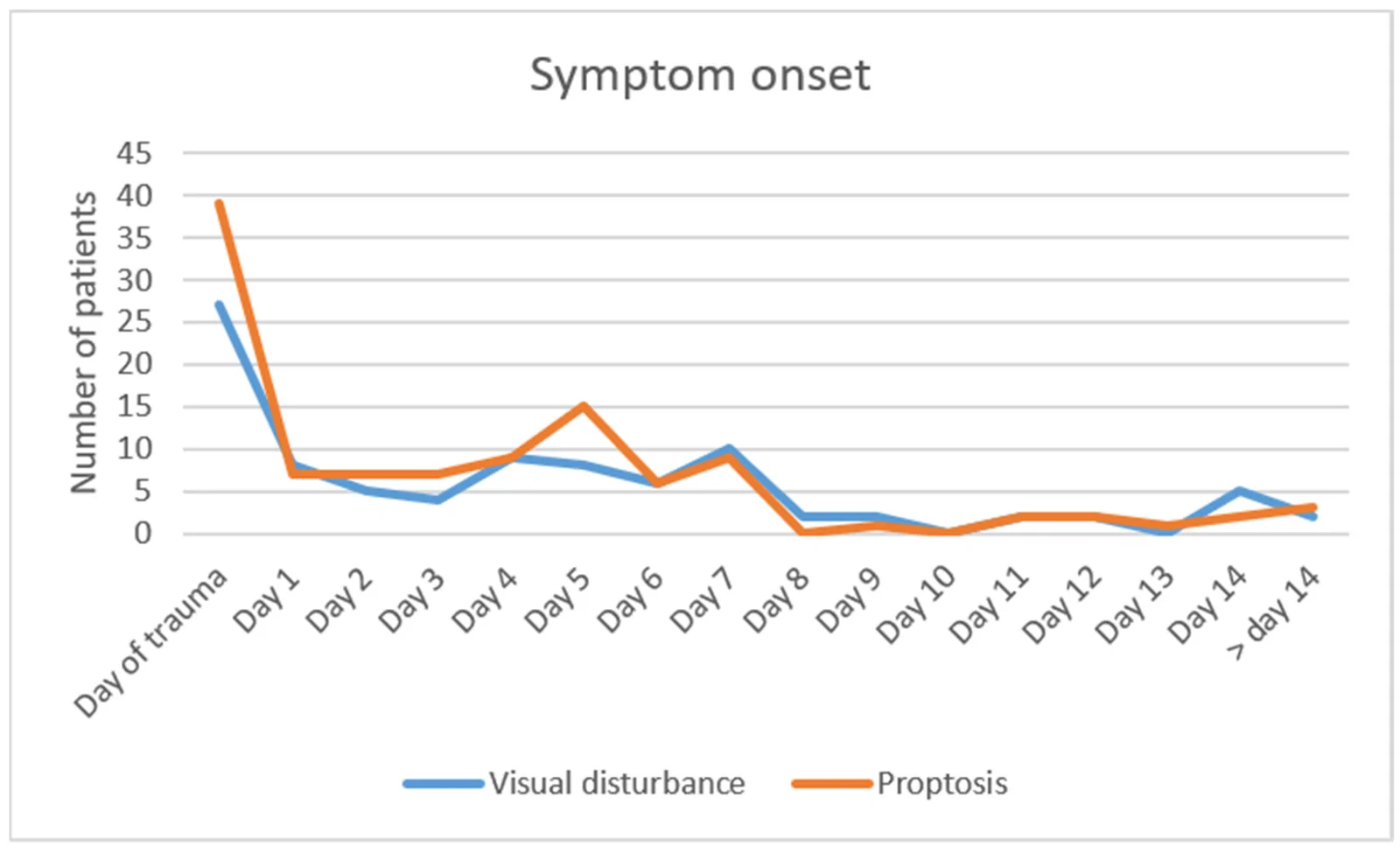
Time to onset of the two most common symptoms of patients with OCS.

**Figure 7:**
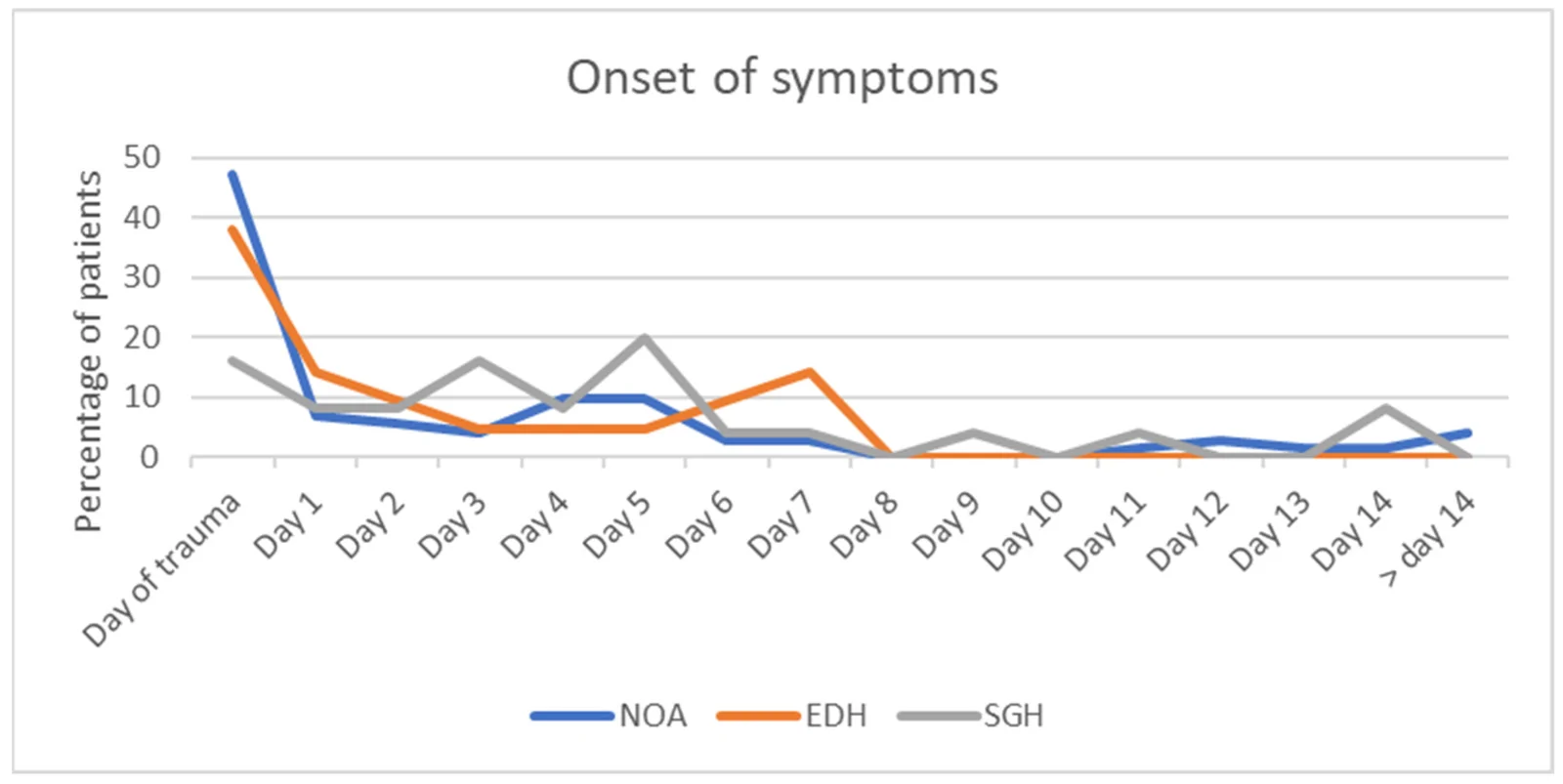
Time to onset of symptoms according to subgroups. NOA = no associated extraorbital hematoma; EDH = assoc. epidural hematoma; SGH = assoc. subgaleal hematoma.

The most common presenting symptoms were proptosis, visual disturbances, displacement of the eye, gaze restriction, pain, and/or a palpable mass within the orbit; additionally, some studies stated if RAPD (relative afferent pupillary defect) was present or not. Many patients presented with a combination of these symptoms. A detailed breakdown of the prevalence of each symptom is provided in Figure 5. Notably, all cases of eye displacement were either downward or downward–out. Of the patients with gaze restriction, 45 were ophthalmoplegic, while the remaining patients had restriction in one direction, most commonly upward trauma” or “day X after trauma.” Detailed timeframes, such as hours to symptom onset or time to presentation in hours, were rarely specified, limiting the ability to conduct further statistical analysis. Proptosis was usually the first symptom, followed by visual disturbances, which were also the most common reasons for seeking medical attention. Interestingly, two distinct peaks in symptom onset were observed: one on the day of the trauma and another between days 4 and 7 post-trauma (Figure 6). This pattern was further elucidated when the onset of symptoms was assessed by subgroup (i.e., no associated extraorbital hematoma, associated epidural hematoma, and associated subgaleal hematoma). Patients with subgaleal hematoma exhibited a significantly later onset of symptoms and presented later for consultation compared to patients without extraorbital hematoma or those with epidural hematoma (*p* < 0.05) (Figure 7).

Mean time to symptom onset and mean time to consultation are summarized in Table 2 There was no significant difference in the time to consultation between patients who developed persistent visual deficiencies and those who did not (*p* = 0.51).

### 3.6. Diagnostics

**Figure 8:**
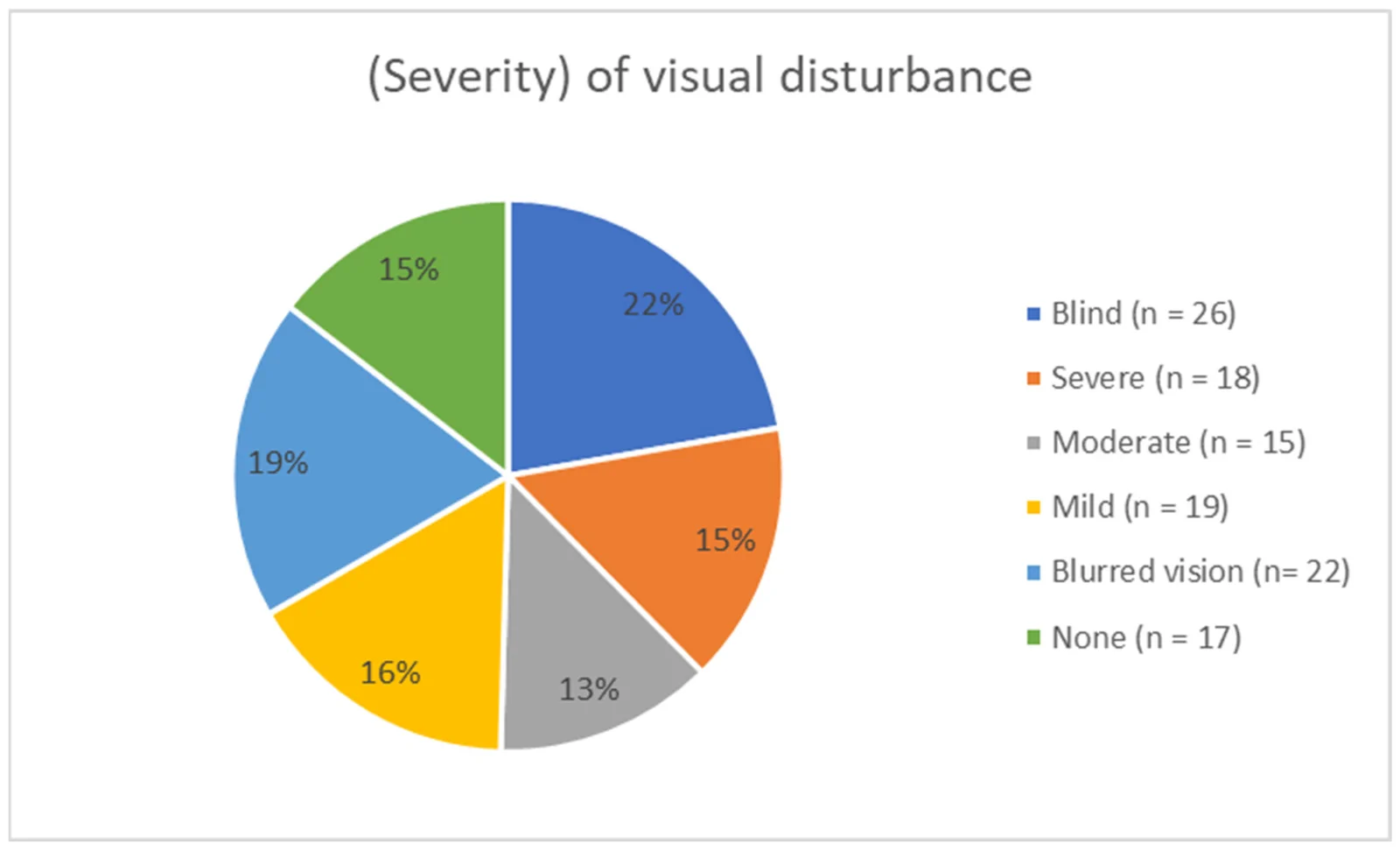
Findings of the clinical examination. Information was available for 102 patients (117 eyes). Visual disturbance severity was defined according to the World health organization (WHO) as follows: blind = VA < 20/400; severe = VA 20/400–20/200; moderate = VA 20/200–20/60; mild = VA 20/60–20/40.

Diagnosis was established through clinical examination in three patients; all other patients underwent additional diagnostic imaging, including sonography (n = 9), head or orbital radiography (n = 11), arteriography (n = 4), CT (computed tomography; n = 110), or MRI (magnetic resonance imaging; n = 18). Clear indications as to why one of the diagnostic tests was chosen above the others were not given in the included studies. Clinical findings were documented in 102 patients (117 eyes), as depicted in Figure 8 [103]. Patients without visual disturbance had at least two other symptoms (as previously outlined) that prompted the diagnosis of OCS.

Intraocular pressure measurements were available for 24 patients (27 eyes). These measurements were normal in 10 patients. The mean intraocular pressure for the 17 patients with pathological readings was 24 mmHg (range 22–70 mmHg).

### 3.7. Therapy

**Figure 9:**
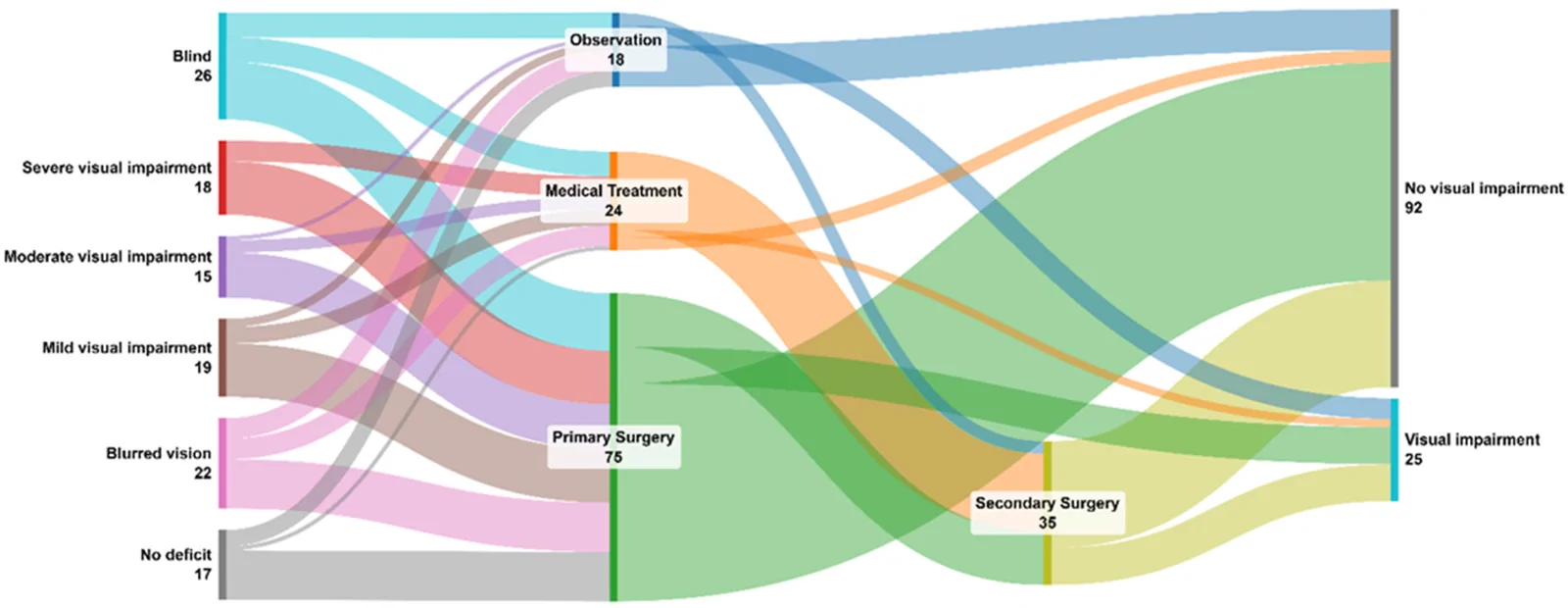
Sankey diagram of the 117 eyes of 102 patients for whom clinical examination results were given in the included studies.

The treatment algorithm for the 102 patients (117 eyes) for whom clinical findings were available is presented in Figure 9. Eighteen eyes underwent an initial trial of observation. As shown in Figure 9, observation was attempted irrespective of the presence of visual disturbance, though most eyes exhibited only blurred vision or no deficit. Three patients (three eyes) required surgery after the initial observational period due to worsening symptoms. Among the eyes initially observed, seven exhibited deficits by the end of the follow-up period. Visual deficits ranged from bilateral blindness in two patients to blindness in one eye in one patient and moderate visual impairment in two patients.

Twenty-four eyes underwent a trial of medical treatment, irrespective of visual impairment. Medical treatment included acetazolamide, steroids, and timolol, or combinations of these agents. Seven of these eyes required surgery due to worsening symptoms. At the end of follow-up, nine eyes experienced visual impairment, ranging from blindness in three eyes (bilateral involvement in one patient) to severe or moderate impairment in six patients.

**Figure 10:**
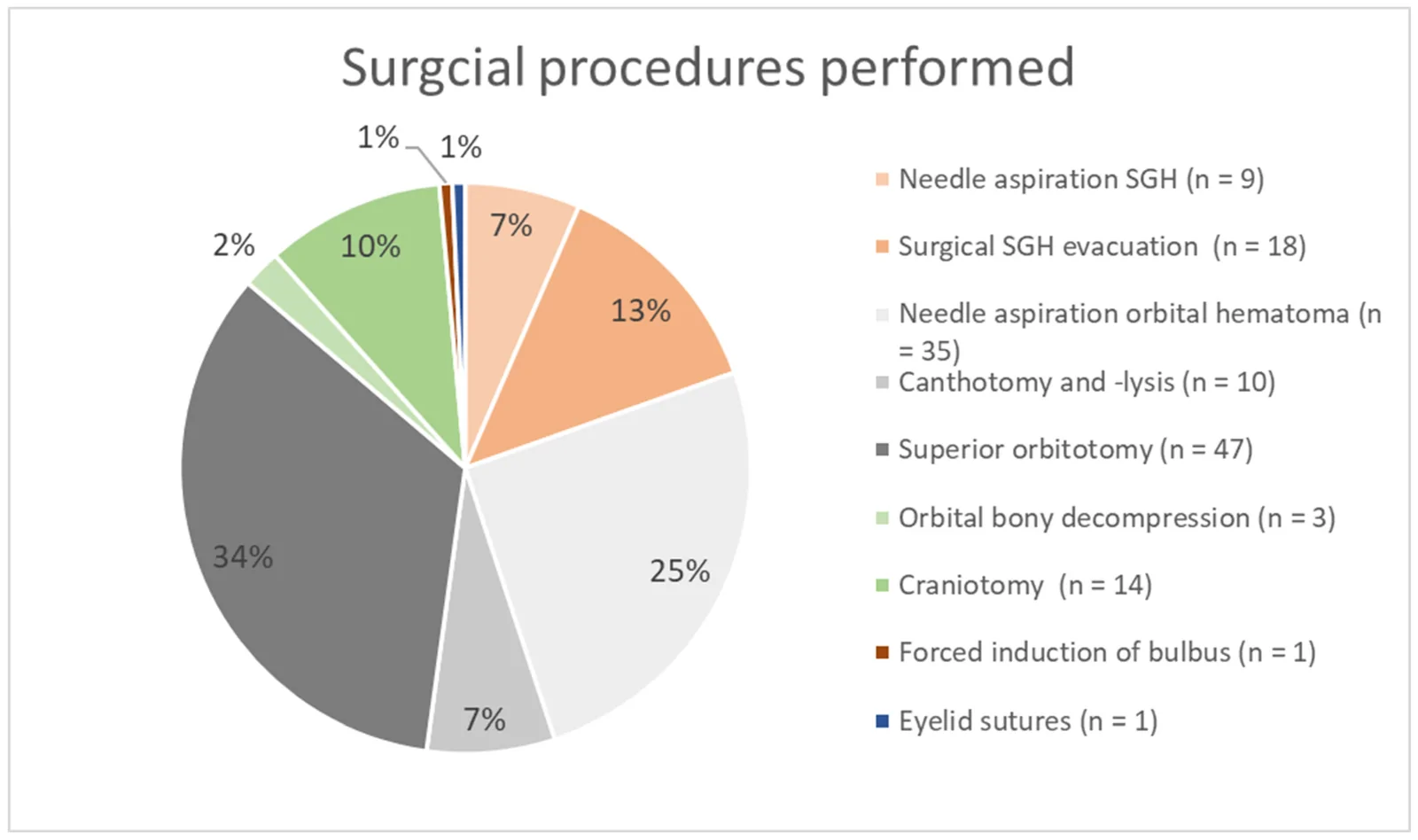
Number of surgical procedures performed either as first-line or second-line procedure in all 103 patients that were treated surgically.

A total of 103 patients (110 eyes) underwent surgical intervention, with 70 patients (75 eyes) receiving primary surgery. The types of surgical procedures performed are outlined in Figure 10. In total, 138 procedures were conducted on the 103 surgically treated patients. A variety of procedures were employed, some tailored to address the associated hemorrhage, such as needle aspiration of the subgaleal hematoma or craniectomy in the case of concurrent epidural hematoma. Other procedures aimed to relieve orbital pressure directly, including needle aspiration of the orbital hematoma, canthotomy and cantholysis, and superior orbitotomy. Among the 80 patients with no associated extraorbital hemorrhage, only 3 underwent a second procedure. Initial treatment was either needle aspiration of the orbital hematoma or canthotomy and cantholysis, with secondary procedures being orbitotomy. Patients with associated subgaleal hematoma underwent a second procedure in 68% of cases (17 out of 25). Initial treatments often focused on aspiration or evacuation of the subgaleal hematoma, leading to temporary symptom resolution. However, in 68% of cases, symptoms recurred, necessitating a more formal approach (orbitotomy or needle aspiration of the orbital hematoma in combination with (re-)evacuation of the subgaleal hematoma). The majority of patients with subperiosteal hematoma (OSH; 102 patients) were treated with either needle aspiration (n = 29) or orbitotomy (n = 43). Both procedures yielded a similar rate of success (95%). Second-line procedures were either a second needle aspiration or bony decompression or cantothomy and cantholysis. Patients with associated epidural hematoma underwent either needle aspiration or orbitotomy, followed by craniectomy. In one case, the epidural hematoma was found to have resolved by the time of craniectomy, most likely having been drained during the needle aspiration of the orbital hematoma.

### 3.8. Outcome

The mean follow-up period was 109 days (SD = 221). At the end of the follow-up period, 21 patients (25 eyes) had sustained visual impairment. Nine eyes (bilateral involvement in two patients) became blind, as assessed by visual acuity. Nine eyes experienced severe visual impairment, five had moderate impairment, and two had mild impairment. Since the time to surgery was recorded similarly to the time to symptom onset, no significant correlation was found between time to surgery and visual outcome (*p* = 0.23). However, patients who underwent first-line surgery had significantly better visual outcomes than those who underwent secondary surgery (*p* < 0.05). Furthermore, of all patients that presented with or developed RAPD (n = 15), ten had visual deficit despite uniformly undergoing timely surgical treatment.

### 3.9. Risk of Bias, Level of Evidence and Publication Bias

The Joanna Briggs checklist for case reports and case series was used to assess for missing data in the eligible studies. If the quality was deemed poor or fair, the study was excluded. Visual acuity testing was used to describe visual outcome. However, the specific method of testing was not stated in the reports, which can, at least in theory, bias the comparability of visual outcomes. However, a recent study showed good agreement between the four most used visual acuity tests [104]. This finding limits the risk of possible bias. Level of evidence was IV. Since OCS is scarce in children, there is an inherent risk of publication bias due to the limited strength of the case load and, therefore, the strength of studies concerning this disease.

## 4. Discussion

This systematic review included 126 children (138 affected eyes) and was predominantly based on case reports and small case series, limiting the certainty of evidence and precluding causal inferences. However, given the rarity and sight-threatening nature of pediatric orbital compartment syndrome, a systematic synthesis of published cases currently represents the highest level of evidence available. Therefore, the findings of this review should be interpreted with appropriate caution, particularly with regard to the apparent differences between pediatric and adult OCS and associations between treatment strategies and outcomes.

Males were affected four times more often than females. As all injuries occurred during recreational activities, the male predominance may be attributed to higher-risk behavior among boys, as previously described in the literature [105]. In contrast to adults, where diffuse retrobulbar hemorrhage is the typical cause of OCS, we found that 81% of children had a localized subperiosteal hematoma, most commonly on the orbital roof.

Likely due to reporting bias (see above), no significant difference was found between patients with permanent visual impairment and those without, based on time to consultation (*p* = 0.51). Nevertheless, animal studies and adult data suggest the retina tolerates 90–120 min of ischemia before irreversible damage occurs [2,3,4]. This threshold is most likely applicable to children as well.

Only in three cases was diagnosis established based on the clinical picture alone; all others underwent additional imaging, 110 of which were CT scans, to confirm the diagnosis before commencing OCS treatment. This is concerning for two reasons: first, delays in treatment—shown to worsen outcomes in adults—may have occurred. Moreover the presence of RAPD appeared to be associated with persistent visual impairment and may therefore represent an important clinical and prognostic indicator in pediatric OCS. The need for urgent treatment is underscored by the widespread understanding that advanced prehospital providers should be familiar with and able to perform a therapeutic measure, e.g., canthotomy and cantholysis [106,107,108,109]. Second, all patients presented with GCS scores of 14–15 and symptoms limited to the orbit, raising questions about adherence to pediatric CT guidelines such as PECARN [106]. Another diagnostic tool that is worth mentioning is sonography. Bedside orbital ultrasonography, performed in nine cases, may offer a radiation-free, time-efficient diagnostic alternative. Ultrasound (US) is a valuable imaging modality for the evaluation of the orbit [107]. It can demonstrate secondary anatomical signs associated with elevated intraorbital pressure, such as globe tenting (“guitar pick” sign), and show retrobulbar/subperiosteal hematoma [108,109,110,111]. Moreover, it can guide needle aspiration—reported to have a 95% success rate for symptom relief in OSH [12,78,84,112]. Given that OSH was the predominant etiology of pediatric OCS, this modality could be highly valuable.

Most patients initially managed with observation or medical therapy eventually required rescue surgery or experienced visual deficits. This supports the need for early surgical intervention in pediatric OCS [2,113]. Patients who were observed and retained normal vision had no visual deficit at initial presentation. Given the severe consequences of OCS, a low threshold for surgical intervention is acceptable and, some argue, even desirable to avoid missed cases [113]. The high frequency of needle aspiration and superior orbitotomy, and the comparatively low use of canthotomy and cantholysis, reflect the predominance of OSH. A tailored approach—e.g., needle aspiration or orbitotomy—may be more effective in such cases as they address both intraorbital pressure and the space-occupying lesion [13,82]. This also reduces the risk of complications, such as calcification of the hematoma [82].

Adult data clearly demonstrate better outcomes with earlier intervention [4,114]. This is consistent with our finding that patients undergoing primary surgery had significantly better visual outcomes than those receiving secondary surgery after failed conservative management (*p* < 0.05). Notably, vision restoration is possible even when symptom onset occurred over two hours prior, underscoring the need for intervention regardless of delay [7,75].

### 4.1. Subgroup Analysis

#### 4.1.1. Newborns

Eight newborns, including one with bilateral OCS, were identified—all cases caused by OSH [9,12,31,44,55,56,88]. Five had an obvious history of traumatic delivery (e.g., prolonged labor, forceps/vacuum assistance, shoulder dystocia), while three did not. In neonates, the loosely attached periosteum can be stripped from the bone during delivery or skull molding, making OSH possible even without obvious trauma. If a newborn presents with exophthalmos, OSH-induced OCS should be suspected regardless of delivery history [59]. Symptom onset ranged from immediately postnatal to one day. In addition to clinical signs (e.g., exophthalmos, globe displacement, RAPD), ultrasonography—used in four cases—successfully aided diagnosis [12]. Surgery was performed in four cases, all involving RAPD; patients with isolated proptosis were observed. Orbitotomy was the most common intervention (n = 3). One patient received lid sutures after unsuccessful medical treatment and remained bilaterally blind, while the others had no visual deficits. These findings stress the importance of timely surgical management. Ultrasound offers a radiation-free option for diagnosis and therapeutic guidance [12].

#### 4.1.2. OCS with Associated Epidural Hematoma

Twenty-one patients had OCS with concomitant EDH, most of whom (n = 15; *p* < 0.05) also had orbital fractures. Road traffic accidents were more common in this subgroup. Symptom onset showed a bimodal distribution: 40% presented within one day, while one-third developed symptoms 4–7 days post-trauma. This delay may be due to blood migration through fractures into orbital spaces [52].

Most patients (n = 19) had OSH; the remainder had retrobulbar hemorrhage. All patients underwent either CT or MRI due to the clinically apparent fracture or trauma mechanism. All received surgical treatment, including craniotomy (alone or combined with orbitotomy) or needle decompression. One patient who initially received needle decompression had complete EDH resolution by the time of craniotomy, highlighting the anatomical connection between EDH and OSH [52,85]. In selected patients without immediate indications for a CT, ultrasound-guided decompression followed by MRI may reduce radiation exposure and surgical invasiveness [12,52,85]. None of the patients in this group had visual impairment at follow-up, likely due to prompt recognition and intervention.

#### 4.1.3. OCS Associated with Subgaleal Hematoma

Twenty-five patients had SGH in addition to OCS. Bilateral eye involvement and coagulopathy were significantly more common in this group (*p* < 0.05); therefore, assessment of the coagulation status in these patients is mandatory. The pathomechanism likely involves shearing forces causing SGH, which then dissects through the loose connective tissue attachments at the arcus marginalis to form an OSH [15,16,46,48,78]. Even minimal trauma, such as hair-pulling or braiding, was sufficient in some cases. Symptom onset and presentation were significantly delayed compared to other groups (each *p* < 0.05), consistent with the time required for hematoma expansion, pressure generation and dissection through the arcus marginalis. CT was performed in 23 of 25 cases, including those with only minor trauma, though clear indications were often lacking. Initial surgical management in over half the cases (n = 14) involved SGH evacuation alone, which often proved insufficient or led to recurrence. This subgroup had a significantly higher rate of secondary procedures, underscoring the need for complete drainage of both SGH and OSH. Four patients had visual deficits at follow-up (three with blindness, one with mild impairment). While symptom onset was typically on the same day as presentation, specific timelines in hours were not reported.

**Figure 11:**
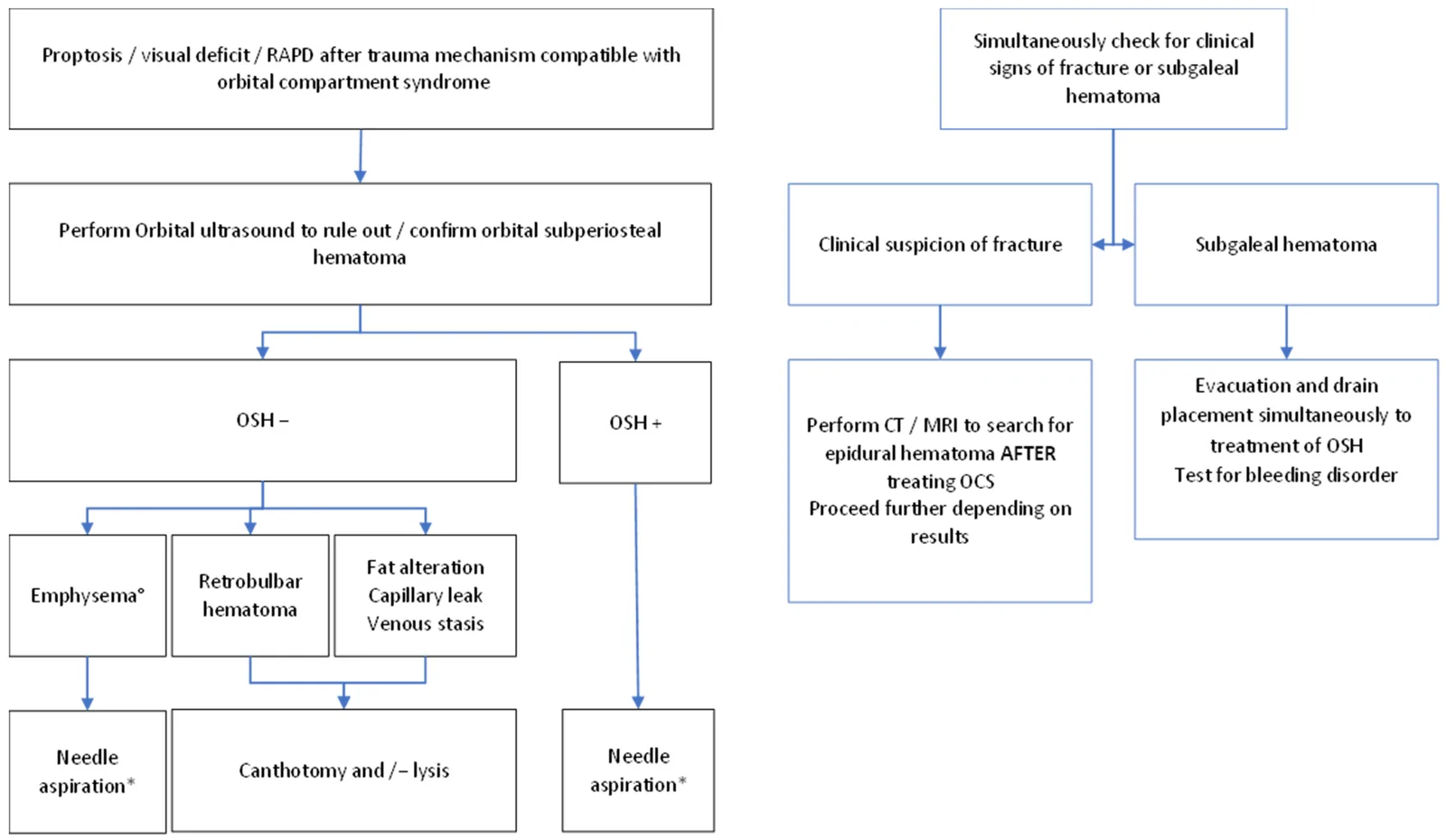
Proposed pragmatic algorithm for the management of pediatric orbital compartment syndrome in children. OSH: orbital subperiosteal hematoma, CT: computed tomography; MRI: magnetic resonance tomography; ° can be identified by trauma mechanism and history; * if failed to relieve symptoms, perform C/CL.

These subgroup findings informed the adaptation of Erickson et al.’s evidence-based algorithm [114] to reflect age-specific pathophysiology, diagnostic considerations, and treatment strategies needed in children (see Figure 11). The proposed algorithm has not been prospectively validated and should therefore be interpreted as expert and literature-informed guidance.

## 5. Strengths and Limitations

One of the most important strengths of this systematic review is that the literature search was undertaken without language limitations, which broadened the included studies and increased the patient collective available for analysis.

The most important limitation of this review is that the available evidence consisted almost exclusively of case reports and small case series. Consequently, the certainty of evidence is low and causal inferences cannot be established. Selection bias, publication bias, and incomplete reporting are inherent limitations of the available literature. Nevertheless, given the rarity and emergency nature of pediatric OCS, the systematic synthesis of published cases currently represents the highest level of evidence available.

Another prominent limitation is that onset of symptoms and time of consultation were usually given as either “on the day of trauma” or “day x after trauma”. Seldom were the hours of symptom onset or time to presentation in hours given, which further affected the statistical analysis.

A further strength is that despite the considerable amount of case reports published more than 50 years ago, the authors were able to retrieve almost all papers deemed eligible for inclusion. This was able thanks to the different but complementary journal subscriptions at the authors’ hospitals.

## 6. Conclusions

This systematic review highlights that the epidemiology, underlying pathophysiology, and clinical presentation of orbital compartment syndrome in children differ substantially from those in adults. Pediatric OCS is more frequently associated with subperiosteal hematomas and distinct trauma mechanisms, often requiring alternative management strategies. The findings suggest that pediatric OCS may require pediatric-specific diagnostic and management considerations. However, these observations should be interpreted cautiously and prospective multicenter studies are needed to further refine management recommendations. Further prospective studies are warranted to validate these subgroup-specific treatment pathways and refine pediatric OCS management guidelines.

## Figures and Tables

**Table 1:** Type pf space occupying lesion concerning subgroups.

Frequency of Each Underlying Pathology Within the Subgroups				
	Orbital subperiosteal hematoma	Emphysema	Retrobulbar hematoma	Fat alteration, capillary leak, venous stasis
NOA	53	9	5	5
SGH	22	1	2	0
EDH	2	19	0	0
Newborns	8	0	0	0

**Table 2:** Time to onset of symptoms and consultation according to subgroups. NOA = no associated extraorbital hematoma; EDH = assoc. epidural hematoma; SGH = assoc. subgaleal hematoma.

	NOA	EDH	SGH
Time to first symptom (days) [mean, SD]	1.9 [3, 1]	3.3 [2, 6]	5.4 [5, 1]
Time to consultation (days) [mean, SD]	2.3 [4, 3]	4.6 [1, 0]	15.4 [2, 9]

## Data Availability

The raw data supporting the conclusions of this article will be made available by the authors on request.

## Supplementary Materials

The following supporting information can be downloaded at: https://www.mdpi.com/article/10.3390/children13080982/s1, Search Terms; Table S1: List of included studies.

## Abbreviations

RAPD	Relative afferent pupillary defect
OCS	Orbital compartment syndrome
OSH	Orbital subperiosteal hematoma
EDH	Epidural hematoma
SGH	Subgaleal hematoma
NOA	No association with extraorbital hematomas
WHO	World Health Organization
US	Ultrasound
CT	Computed tomography
MRI	Magnetic resonance imaging
