# Supplementary material for: Traumatic Orbital Compartment Syndrome in Children—Systematic Review and Descriptive Summary
Source: Children (Basel). 2026 Jul 24;13(8):982. doi: 10.3390/children13080982 (PMC13511678; doi:10.3390/children13080982)
Supplement: Supplementary file 1 [file children-13-00982-s001.zip › children-4379908-supplementary.pdf]

SUPPLEMENTS

1. SEARCH TERMS

PubMed

(orbital emphysema or canthotomy or orbital compartment syndrome or orbital compression syndrome or retrobulbar hematoma or retrobulbar hemorrhage or subperiost* orbital hematoma) and trauma and (infan* or newborn* or new-born* or perinat* or neonat* or baby or baby* or babies or toddler* or minors or minors* or boy or boys or boyfriend or boyhood or girl* or kid or kids or child or child* or children* or schoolchild* or schoolchild or school child or school child* or adolescen* or juvenil* or youth* or teen* or under*age* or pubescen* or pediatrics or pediatric* or paediatric* or peadiatric* or school or school* or prematur* or preterm*)

EMBASE

('orbital emphysema'/exp OR 'orbital emphysema' OR (('orbital'/exp OR orbital) AND ('emphysema'/exp OR emphysema)) OR 'canthotomy'/exp OR canthotomy OR 'orbital compartment syndrome'/exp OR 'orbital compartment syndrome' OR (('orbital'/exp OR orbital) AND compartment AND ('syndrome'/exp OR syndrome)) OR 'orbital compression syndrome'/exp OR 'orbital compression syndrome' OR (('orbital'/exp OR orbital) AND ('compression'/exp OR compression) AND ('syndrome'/exp OR syndrome)) OR 'retrobulbar hematoma'/exp OR 'retrobulbar hematoma' OR (retrobulbar AND ('hematoma'/exp OR hematoma)) OR 'retrobulbar hemorrhage'/exp OR 'retrobulbar hemorrhage' OR (retrobulbar AND ('hemorrhage'/exp OR hemorrhage)) OR 'subperiost* orbital hematoma' OR (subperiost* AND ('orbital'/exp OR orbital) AND ('hematoma'/exp OR hematoma))) AND ('trauma'/exp OR trauma) AND ((infan* OR newborn* OR 'new born*' OR perinat* OR neonat* OR 'baby'/exp OR baby OR baby* OR babies OR toddler* OR 'minors'/exp OR minors OR minors* OR

'boy'/exp OR boy OR boys OR boyfriend OR boyhood OR girl* OR kid OR kids OR 'child'/exp OR child OR child* OR children* OR schoolchild* OR 'schoolchild'/exp OR schoolchild OR 'school child'/exp OR 'school child' OR (('school'/exp OR school) AND ('child'/exp OR child)) OR 'school'/exp OR school) AND child* OR adolescen* OR juvenil* OR youth* OR teen* OR under*age* OR pubescen* OR 'pediatrics'/exp OR pediatrics OR pediatric* OR paediatric* OR peadiatric* OR 'school'/exp OR school OR school* OR prematur* OR preterm*)

<u>Web of Science</u>

(orbital emphysema or canthotomy or orbital compartment syndrome or orbital compression syndrome or retrobulbar hematoma or retrobulbar hemorrhage or subperiost* orbital hematoma) and (infan* or newborn* or new-born* or perinat* or neonat* or baby or baby* or babies or toddler* or boy or boys or boyfriend or boyhood or girl* or kid or kids or child or child* or children* or schoolchild* or schoolchild or school child or school child* or adolescen* or teen* or pediatrics or pediatric* or paediatric* or peadiatric* or prematur* or preterm*)

2. Table S1: LIST OF INCLUDED STUDIES

Title	Authors	Publication Year	Language	No. of pts.	Subgroup
Subgaleal haematoma extending into the orbit following blunt head trauma a a cause of permanent blindness: A case illustrated review	Adeleye	2017	English	1	SGH (subgaleal hematom a)
Subperiosteal hematoma of the orbit associated with subfrontal hematoma presenting as proptosis	Agrawal	2007	English	1	EDH (epidural hematom a)
Giant cephalhematoma in a 15-year-old boy. Unilateral amaurosis as the main complication	Aguas	1995	English	1	SGH
Urgent subconjunctival needle decompression for orbital compartment Emphysema caused by compressed air injury	Akbari - Kamarani	2020	English	1	NOA (no associate d extraorbit al hematom a)
Visual recovery following immediate decompression of traumatic retrobulbar hemorrhage via transcranial approach	Amagasaki	1998	English	1	NOA
Orbital Subperiosteal Hematoma in the Newborn Causing Unilateral Proptosis: Ultrasound as First-Line Imaging Modality	Angkasa*	2017	English	2	2 x NOA
Orbital, Periorbital, and Intracranial Emphysema Caused by Compressed Air Injury in a 5-Year-Old Child	Bagheri	2018	English	1	NOA
Epidural hematoma revealed by exophthalmia, two cases reports	Bahloul	2009	English	2	2 x EDH
Visual loss following traumatic asphyxia in children	Baldwin	1988	English	2	2 x NOA
Orbital hemorrhage following extracorporeal membrane oxygenation in a newborn	Bart*	1997	English	1	NOA
Posttraumatic subperiosteal orbital hematoma revealed by ocular motility impairment	Belghith	2015	Frensh	1	NOA

Diagnosis and treatment of orbit posttraumatic subperiosteal hemorrhage in a child, associated with a subdural intracranial hemorrhage	Brucoli	2005	English	1	NOA
Surgical management of posttraumatic intraorbital hematoma	Brucoli	2012	English	1	NOA
Visual recovery after evacuation of orbital emphysema	Chaudhry	2007	English	1	NOA
Subperiosteal hematoma of the orbit: case report	Chaves	2007	Portuguese	1	NOA
Management of acute traumatic retrobulbar haematomas: a 10-year retrospective review	Chen	2012	English	2	2 x EDH
Medial Wall Fracture and Orbital Emphysema Mimicking Inferior Rectus Entrapment in a Child	Collin	2015	English	1	NOA
Orbital subperiostal hematoma associated with extradural hematoma: one case report	Compaore	2011	French	1	EDH
Subperiosteal hematoma of the orbit	Dhir	1982	English	1	NOA
Pediatric posttraumatic orbital subperiosteal hematoma	Eshraghi	2021	English	5	5 x NOA
A marked exophthalmos and corneal ulceration caused by delayed massive expansion of a subgaleal hematoma	Fujisawa	2005	English	1	SGH
Traumatic subperiosteal hematoma of the orbit: a report of 2 cases	Ganesan	2008	English	2	2 x NOA
Subperiosteal orbital hemorrhage as initial manifestation of Christmas disease (factor IX deficiency)	Guirgis	2002	English	1	SGH
Traumatic subfrontal extradural hematoma presenting with proptosis	Gupta	2010	English	1	EDH
Huge recurrent subperiosteal hematoma in a 9-year-old boy: case report with special reference to pathologic and anatomic studies	Higashi	2008	English	2	2 x SGH
Subperiostal hematoma of the orbit	Horaczek	1983	German	1	NOA
Orbital compartment syndrome in a burn patient without aggressive fluid resuscitation	Hurst	2014	English	1	NOA

Neonatal orbital subperiosteal haematoma following shoulder dystocia	Javaid*	2015	English	1	NOA
Orbital Compartment Syndrome Following Post-Traumatic Subgaleal Hematoma	Jenkins	2017	English	1	SGH
Subperiosteal Orbital Extension of Subgaleal Hematoma: A Case Report.	Jeong	2000	Korean	1	SGH
Ocular involvement after subgaleal hematoma	Karcioglu	2008	English	1	SGH
Orbital subperiosteal hematoma (epidural hematoma of the orbit)	Katz	1981	English	1	NOA
Subperiosteal orbital hematoma: visual recovery following delayed drainage	Kersten	1987	English	1	NOA
Delayed Massive Expansion of Subgaleal Hematoma Complicated with Proptosis in Hemophilia B	Kim	2011	English	1	SGH
Clinical features and management of posttraumatic subperiosteal hematoma of the orbit	Kim	2021	English	4	3 x NOA, 1 x EDH
Ocular changes following air-blast injury	King	1971	English	1	NOA
Rapid dissapearance of acute epidural hematoma by percutaneous aspiration of subperiosteal hematoma	Kinoshita	2007	Japanese	1	EDH
Traumatic eye and intracranial air-movement from a subconjunctival to an intracranial position	Koenig	1977	English	1	NOA
Childhood subgaleal hematoma following minor head trauma	Kuban	2002	English	1	SGH
Bilateral exophthalmos in the newborn	Kundert*	1983	English	1	NOA
Subperiosteal hematoma of the orbits following forceps delivery--a case report	Kurleman*	1951	German	1	NOA
Subperiosteal hematoma of the orbit: case presentation	Landa	1998	English	1	NOA
Proptosis from a cephalhematoma in a twelve-year-old girl--a case report	Lee	1988	English	1	SGH
Bilaterl proptosis from a subgaleal hematoma	Lee	1991	English	1	SGH
Posttraumatic subperiosteal orbital hematoma	Leovic	2009	English	1	NOA

Retrobulbar hemorrhage: a case report	Machado	2006	English	1	NOA
Proptosis and diplopia following traumatic asphyxia	Macnab	1987	English	1	NOA
Acute Subperiosteal Hematoma of the Orbit with Visual Impairment: An Unconventional Presentation	Maithani	2016	English	1	NOA
Subacute phase treatment of subperiosteal hematoma of the orbit with epidural hematoma in the frontal cranial fossa: case report	Mikami	2012	English	1	EDH
Emergency surgical decompression of the orbit may rescue impaired vision even in a patient presenting late with a retrobulbar haematoma: A case report from rural southwestern Uganda	Mugarura	2022	English	1	NOA
Post-traumatic orbital sub periosteal hematoma in children: Clinical spectrum and management outcomes	Mukherjee	2022	English	6	6 x NOA
Orbital Hemorrhage in a Newborn	Munoz*	1989	English	1	NOA
Subperiosteal hematoma of the orbit associated with subfrontal extradural hematoma	Naja	2002	English	1	EDH
Posttraumatic subgaleal and orbital hematoma due to factor XIII deficiency	Natarajan	2011	English	1	SGH
Pediatric orbital emphysema caused by a compressed-air pistol shot: a case report	Navarro - Mingorance	2013	Spanish	1	NOA
Concomitant occurrence of subfrontal extradural hematoma and orbital subperiosteal hematoma: a rare entity	Nayak	2010	English	3	3 x EDH
Subperiosteal intraorbital haematoma following minor head trauma. Case report	Pasaoglu	1989	English	1	SGH
Subgaleal hematoma with delayed proptosis and corneal ulceration	Pomeranz	1995	English	1	SGH
Post-traumatic subgaleal hematoma with subperiosteal orbital extension	Pope-Pegram	1986	English	1	NOA
Bilateral proptosis from a subgaleal hematoma	Prakash	1990	English	1	SGH

Subperiosteal hematoma and orbital compression syndrome following minor frontal trauma in sickle cell anemia: case report	Procianoy	2005	English	1	NOA
Orbital compartment syndrome mimicking cerebral herniation in a 12-yr-old boy with severe traumatic asphyxia	Prodhan	2003	English	1	NOA
Continous B scan ultrasound guided post-traumatic sub-periosteal orbital hematoma drainage: An advantage over routine needle drainage procedure	Pujari	2019	English	1	NOA
Hairpulling causing vision loss: a case report	Puri	2018	English	1	SGH
Needle aspiration of a traumatic subperiosteal haematoma of the orbit	Rojas	2002	English	1	NOA
Ipsilateral Exophtalmos due to subfrontal epidural hematoma	Romano	1983	English	1	EDH
The role of early office-based needle aspiration in the management of subperiosteal hematoma	Sabermoghaddam	2018	English	4	4 x NOA
Subperiosteal hematoma of the orbit with osteoneogenesis	Sabet	2001	English	1	NOA
Rare association of subperiosteal hematoma of the orbit with frontal extradural hematoma: a case report	Sanda	2022	Frensh	1	EDH
Subperiosteal hematomas of the orbit: angiographic and computed tomographic diagnosis	Seigel	1982	English	1	SGH
Emergent Needle Aspiration of an Orbital Subperiosteal Hematoma	Shameer	2016	English	1	NOA
Orbital subperiosteal hematoma associated with subfrontal extradural hematoma	Sharma	2007	English	1	EDH
Bilateral simultaneous orbital hemorrhage and compartment syndrome as the presenting sign of acquired hemophilia A in an 11-year-old girl	Shatnawi	2020	English	1	SGH
Traumatic Orbital Compartment Syndrome: A Sight Threatening Emergency	Singh	2019	English	1	NOA

Clinico-radiological features and treatment outcomes in children with traumatic orbital subperiosteal hematoma	Singh	2018	English	10	10 x NOA
Subperiosteal orbital haematoma presenting as proptosis at birth	Skull*	1997	English	1	NOA
Proptosis as a presenting sign of extradural haematoma	Stewart	1993	English	1	EDH
A Case of Subgaleal Hematoma with Exophthalmos and Diplopia	Takano	2015	Japanese	1	SGH
Ipsilateral Exophtalmos in chrinic extradural fluid-hematoma - case report	Takemura	1980	Japanese	1	EDH
Traumatic optic neuropathy--to treat or not to treat?	Tandon	2009	English	1	NOA
MRI of subperiosteal hematoma of the orbit	Tonami	1994	English	1	NOA
Epidural haematoma and unilateral exophthalmos--a review	Umansky	1989	English	1	EDH
Orbitopalpebral emphysema and traumatic uveitis from compressed air injury	Walsh	1972	English	1	NOA
Exophtalmos and Epidural Hematoma	Watts	1976	English	1	EDH
Fall from bed resulting in emergent lateral orbital canthotomy…7 days later	Wiser	2022	English	1	SGH
Posttraumatic subgaleal hematoma extending into the orbit as a cause of permanent blindness	Wolter	1978	English	1	SGH
Clinical picture and management of subperiosteal hematoma of the orbit	Wolter	1976	English	1	NOA
Posttraumatic subperiosteal hematomas of the orbit in children	Yazici	2011	English	5	5 x NOA
Proptosis after hair pulling	Yip	2003	English	1	SGH
Association of a Subperiosteal Hematoma With Minor Injury	Akiyama	2015	English	1	SGH
